# Minimally Invasive Scoliosis Surgery with Oblique Lateral Lumbar Interbody Fusion: Single Surgeon Feasibility Study

**DOI:** 10.7759/cureus.1389

**Published:** 2017-06-25

**Authors:** Hamid Abbasi, Lynn Miller, Ali Abbasi, Vali Orandi, Kamran Khaghany

**Affiliations:** 1 Tristate Brain and Spine Institute; 2 Minnesota Disc Replacement and Spine Restoration Center; 3 Pritzker School of Medicine, The University of Chicago Medicine; 4 Vascular and Interventional Radiology, Lake Region Healthcare; 5 Radiology, Lakes Regional Healthcare

**Keywords:** scoliosis, deformity surgery, spinal fusion, lumbar spine, spine surgery, operative surgical procedures, minimally invasive surgery, interbody fusion, oblique lateral lumbar interbody fusion

## Abstract

**Background:**

Degenerative deformities of the spine have traditionally been treated with extensive open surgeries. However, these open procedures are associated with a high degree of surgical morbidity. In this study, we explore whether clinical improvement in patients with spinal deformities can be achieved using a new minimally invasive surgery (MIS) called oblique lateral lumbar interbody fusion (OLLIF). OLLIF is a MIS single surgeon procedure in which the disc is approached through Kambin’s triangle. OLLIF can achieve correction of spinal deformities through careful cage placement.

**Purpose:**

The purpose of this study is to establish the safety and efficacy of using OLLIF to correct spinal deformities and to collect early outcome data. Collected data includes perioperative outcomes, patient reported outcomes, and radiographic outcomes.

**Study design/setting:**

This study is a retrospective review of 37 OLLIF surgeries in 36 patients with symptomatic degenerative spinal deformity. Collected perioperative data included surgery time, blood loss, and hospital stay. Follow-up was conducted at least 150 days post surgery. We recorded complications and patient reported outcomes such as Oswestry Disability Index (ODI) and pain scale. Imaging was conducted pre- and post-surgery. Fusion rates and changes in Cobb angle were also measured.

**Results:**

A total of 37 surgeries that treated 100 vertebral levels were performed. For two and three level procedures, respectively, the mean blood loss was 83 and 178 ml, the average surgery time was 74 and 158 minutes and the average hospital stay was 2.6 and 3.3 days. The patients ambulated within 24 hours in all but two cases. The patients reported pain improvements on the ten-point pain scale from 8.3 to 3.7 (p<0.001) and on the ODI from 53% to 32%. Cobb angles decreased from 16° to 9.3° (p<0.001), amounting to 2.5° of correction per level of surgery. Detailed imaging was reviewed by independent radiologists for 24 cases and 100% interbody fusion was achieved along with 71% right posterolateral and 74% left posterolateral fusion. There were three cases of mild nerve irritation/neuropraxia and no infections.

**Conclusions:**

OLLIF is a safe and effective MIS technique to correct adult degenerative scoliosis. Unlike alternative procedures, OLLIF is technically less complex than comparable procedures and can safely be used from the thoracolumbar junction to S1.

## Introduction

Degenerative spine disease is one of the most common causes of disability in an aging population. Mechanical back pain, claudication and radicular symptoms from lumbar spondylosis along with degenerative disc and facet disease lead to reduced mobility and progressive loss of daily living activities and quality of life. In many cases, deformity in the coronal and sagittal plane is central to the mechanisms of pain and disability. Surgical interbody fusion of the symptomatic levels is an effective treatment option to restore alignment and provide indirect decompression of neural elements to relieve radicular symptoms [1-4].

Degenerative adult scoliosis is present in 68% of those over 60 years old but is often asymptomatic [5]. Symptomatic prevalence is identified in 6% of patients over the age of 50. If scoliosis becomes symptomatic, surgical correction is appropriate. Decompression alone can increase the deformity [6-8]. Adding posterior instrumentation to the decompression improves stability but requires longer anesthesia times and hospital stays and increases blood loss [9-12]. Recently, posterior approaches have been complemented by interbody grafts through anterior and lateral approaches to deliver enhanced biomechanical stability [13]. Posterior open procedures often require extensive osteotomies and are limited by the presence of the thecal sac and the necessity for nerve root retraction. These open approaches are further complicated by extensive soft tissue damage to the paraspinal muscles and posterior tension bands, which is associated with a high incidence of surgical morbidity [14].

Minimally invasive spine surgery (MIS) posterior approaches have evolved to substantially reduce disruption of soft tissue. Anterior/lateral approaches minimize retraction of neural structures but limit exposure to the spinal canal, cauda equina, and nerve roots. Anterior approaches also risk abdominal and vascular complications, and both lateral and anterior approaches increase the likelihood of lumbar plexus and psoas muscle damage. Today, no MIS approach is routinely used to correct degenerative scoliosis. In this study, we explore whether a new MIS spinal fusion called oblique lateral lumbar interbody fusion (OLLIF) can overcome the difficulties of previous MIS surgeries to correct degenerative scoliosis.

OLLIF is a recent innovation in MIS spinal fusion that has been used to effectively treat degenerative spine disease and lower back pain. Indications for OLLIF include foraminal stenosis causing radiculopathy, lumbar stenosis causing neurogenic claudication, degenerative lumbar spinal deformity with symptomatic spondylolisthesis, and mild stenosis [15]. OLLIF combines the benefits of minimally invasive approaches without the complications traditionally associated with anterior/lateral MIS approaches. OLLIF is performed with the patient in the prone position and should not be confused with oblique (anterior) lumbar interbody fusion/anterior to psoas (OLIF-ATP/ OALIF), where the approach is through the abdominal cavity in the supine or lateral position and is usually performed with the help of an access surgeon.

We have collected experience with OLLIF in over 500 patients to date. In this study, we describe our experience with a subset of 36 OLLIF patients for whom scoliosis was a primary indication for the surgery. We describe the experience from a technical perspective and report perioperative outcomes and a minimum clinical follow-up of six months post surgery, including fusion rates and radiographic Cobb angles, pre- and post-surgery.

## Materials and methods

### Study design

This study is a retrospective review of degenerative scoliosis patients who underwent an OLLIF procedure performed between March 2012 and October 2016. All procedures were performed by a single surgeon in three Minnesota hospitals: Douglas County Hospital, Alexandria, MN; Riverview Health, Crookston, MN; and Fairview Ridges Hospital, Burnsville, MN. Patients were selected for study participation from an electronic database that includes all OLLIF cases to date. Additional patient data was reviewed from patient files at the institution of surgery and the clinic. The study was approved by the Pearl Institutional Review Board (Indianapolis, IN) in November 2016 (IRB approval number: 16-TRIS-105). Informed consent was waived and no identifying information (such as names or other identifying data) is included in this article.

### Patient selection

In general, patients were eligible for OLLIF if they were diagnosed with lumbar/lumbosacral scoliosis, spondylolisthesis, degenerative disc disease, foraminal stenosis, and non-facet based spinal stenosis and had completed a full course of conservative therapy. Contraindications for OLLIF were osteogenic spinal stenosis, bony obstruction, large facet hypertrophy, Grade II listhesis, and other gross deformities.

OLLIF patients were included in this particular study if their primary diagnosis was scoliosis and their Cobb angle exceeded seven degrees. Patient demographics are shown in Table 1.

**Table 1:** Patient demographics Demographics of the study population. One patient underwent a staged procedure; therefore, the 37 surgeries correspond to 36 patients.

# Levels	# Surgeries	# Male	BMI	Age
1	5	3	26±7	68±13
2	13	10	30±5	72±11
3	12	10	29±9	66±12
4	4	3	30±13	72±7
5	1	1	34±0	72±0
6	2	2	27±5	73±19

### Outcome measures

Anesthesia/surgery times, blood loss, and fluoroscopy times were recorded for all patients by the clinic staff and entered into the electronic medical records (EMR) database immediately after surgery. Facility records were reviewed for postoperative ambulation times, pain scores, Oswestry scores, and length of stay. Because no suction is used in OLLIF procedures, blood loss for the OLLIF group was measured by weighing sponges and subtracting dry weight. Cobb angles were measured by drawing a line parallel to the superior endplate of the most cephalad angulated vertebra and the inferior endplate of the most caudal angulated vertebra considered for the surgery and measuring the angle between the two lines. For bilateral scoliosis, the maximum of the angles in each direction was used. Fusion rates and rates of screw loosening/breach were read by two independent radiologists. Oswestry and pain scale questionnaires were given to the patient at each follow-up and complications were monitored. Neuropraxia was defined as weakness below 3/5. Nerve irritation was defined as paraesthesia, dysesthesia, anesthesia, and weakness down to 4/5.

### Surgical procedure

**Figure 1:**
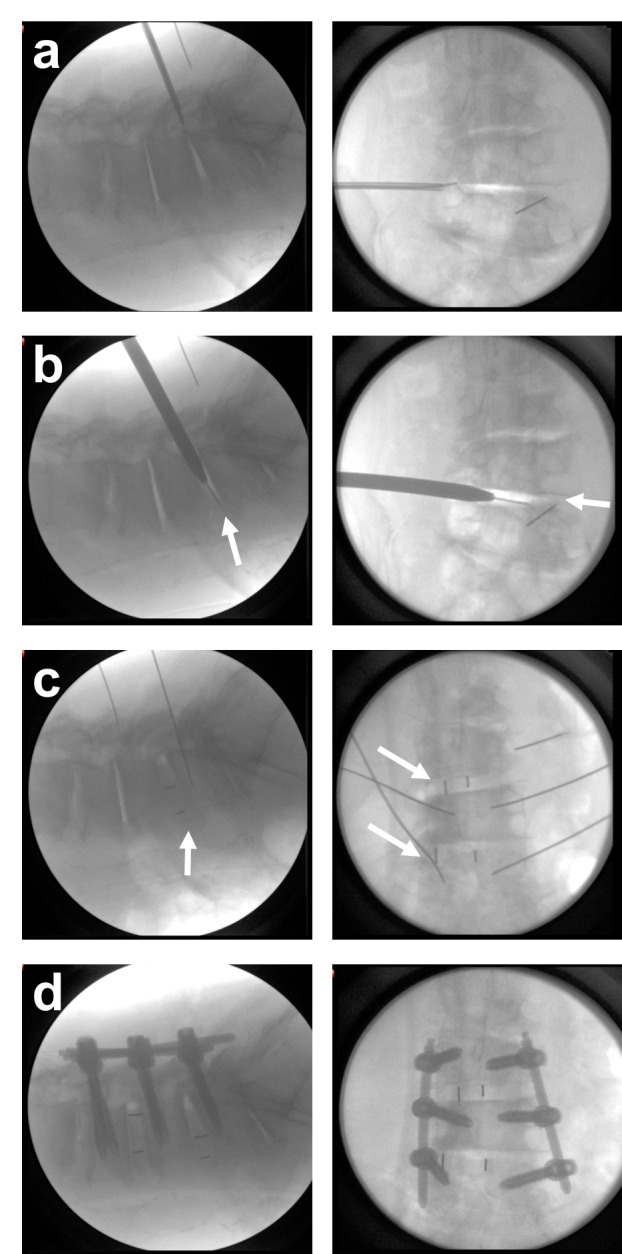
Oblique lateral lumbar interbody fusion deformity correction under fluoroscopic imaging (left lateral view, right anteriorposterior view). a) Approach b) Entry into disc space achieves significant decompression (arrows) c) Cage placement (arrows) d) Procedure is complemented with posterior fixation.

The complete surgical technique for OLLIF is described in Abbasi, et al. [15]. Briefly, the disc is approached through the Kambin’s triangle aided by bilateral fluoroscopy and electrophysiological monitoring. A dilator is inserted over which an access portal (10 mm outside/8 mm inside) is placed. The access portal creates a sealed connection from the skin to the disc. The discectomy is performed entirely through the access portal and the disc space is packed with tricalcium phosphate soaked in autologous bone marrow aspirate. Next, the portal is removed and the cage is inserted under continuous biplanar fluoroscopy and electrophysiological monitoring. Interbody fusion is accompanied by posterior fixation. Due to anatomical considerations in certain cases, a variation of OLLIF called minimally invasive direct lateral interbody fusion (MIS-DLIF) is performed. In MIS-DLIF, the disc space is not accessed through the Kambin’s triangle (posterior to the nerve root), but is approached anterior to the nerve root [16]. The different stages of an OLLIF procedure under biplanar fluoroscopic imaging are displayed in Figure 1.

### OR setup

The patient is placed in the prone position on the operating table. To simplify the approach, the patient is tilted away from the surgeon by 3-5° until after the cage is inserted. Next, bi-planar fluoroscopy is set up. Each level is addressed segmentally.

**Figure 2:**
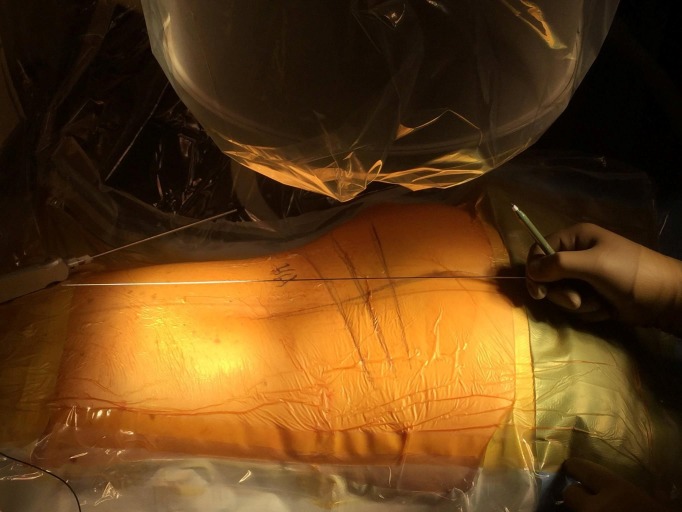
Identifying disc trajectory without alignment of the endplates. The disc trajectories in L3-4, L4-5, L5-S1 are marked. Due to lumbar lordosis, the endplates in all three levels are not in the same plane. Nevertheless, disc trajectory can be identified in the AP view and marked on the skin.

In the lateral view, the endplates of the target level should line up well for cage insertion. In the anteriorposterior (AP) view, the direction of the disc (disc trajectory) needs to be visible but unlike the lateral view, the endplates do not need to be aligned in the AP view. The spinous process should be centered between the pedicles. For scoliosis correction, extra care was given to segmentally align each disc and pedicle following the curvature of the scoliosis. In practice, adjusting the biplanar fluoroscopic imaging for torsion was even more crucial to avoid vital structures anterior to the vertebral body. In the AP view, the posterior patient midline for each horizontal disc trajectory is marked. A vertical line showing the midpoint of each disc is marked in the lateral view. Completed markings are shown in Figure 2.

To find the incision point, the depth of the disc in the lateral view is measured and marked. Next, the midpoint of the disc in the AP view is marked. The incision is located equidistant from the above two marked points. Multiple levels can be approached through the same incision by shifting the skin or approaching the disc at a slight angle. For severe scoliosis or long constructs, locating each segment is paramount and multiple incisions may be necessary. Torsion may require unusual positioning of the C-arm to enable adequate imaging.

**Figure 3:**
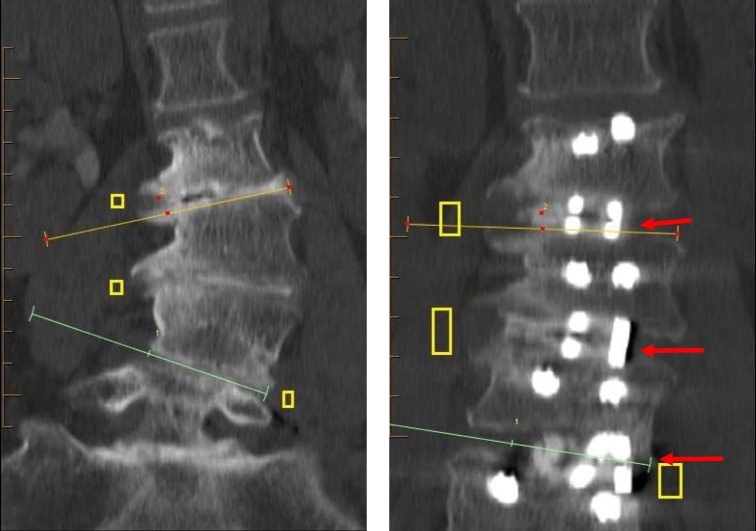
Deformity correction can be achieved through careful cage placement. Pre- and postoperative computerized tomography of an oblique lateral lumbar interbody fusion-deformity correction. Foraminal decompression is achieved by both an increase in the intervertebral disk space and by selecting the laterality of the cage to correct the deformity. Substantial segmental correction can be achieved in this way.

Other than the above considerations, the technical approach to discectomy, endplate preparation, and cage entry are as described in our OLLIF technical paper [14]. The correction of deformity is achieved through careful cage placement: right and left for lateral scoliosis correction and anterior and posterior for lordosis correction. To enable a more horizontal cage placement, the OLLIF approach can be modified to enter the disc space anterior to the nerve root rather than through the Kambin’s triangle, similarly to MIS-DLIF [16]. Some electrophysiological activity is not unusual during cage entry. However, all activity subsides after the cage is placed because the cage increases pedicle distance and causes anatomical/physiological foraminotomy (indirect decompression). A fluoroscopic image of completed cage entry is then taken to confirm positioning. Imaging illustrating the cage after a completed procedure is shown in Figure 3.

After cage placement, all patients undergo percutaneous posterior pedicle screw fixation as described in our technical OLLIF paper [15]. In patients with deformity, special consideration is given to pedicle screw placement because of anatomical variation in the trajectory of the pedicle due to torsion and Cobb angle. We achieve complementary posterolateral fusion through roughening of the facet and the placement of biologics. This “facet de-cortiﬁcation” is achieved by sliding a slit osteotome over the K-wire to roughen the surface of the facet.

## Results

### Perioperative outcomes

Perioperative outcomes are listed in Table 2. Two patients underwent staged procedures, in one case both procedures were OLLIFs, in one case the second stage was an open procedure. Only the OLLIF procedures are included in the outcomes for this study. Therefore, 37 surgeries correspond to a total of 36 patients.

**Table 2:** Perioperative outcomes Perioperative outcomes of the study. Values are mean±standard deviation. Only one patient underwent a five level procedure; therefore, no standard deviation is provided.

# Levels	# Surgeries	Blood Loss (ml)	OR Time (min)	Fluoro Time (s)	Hospital Stay (days)
1	5	116±215	62±40	133±107	3±1.7
2	13	83±73	74±23	374±92	2.6±0.5
3	12	178±103	158±128	599±250	3.3±0.9
4	4	210±152	154±25	594±462	3.8±0.5
5	1	256	157	810	7
6	2	760±622	177±30	330±324	4±0

### Ambulation

**Figure 4:**
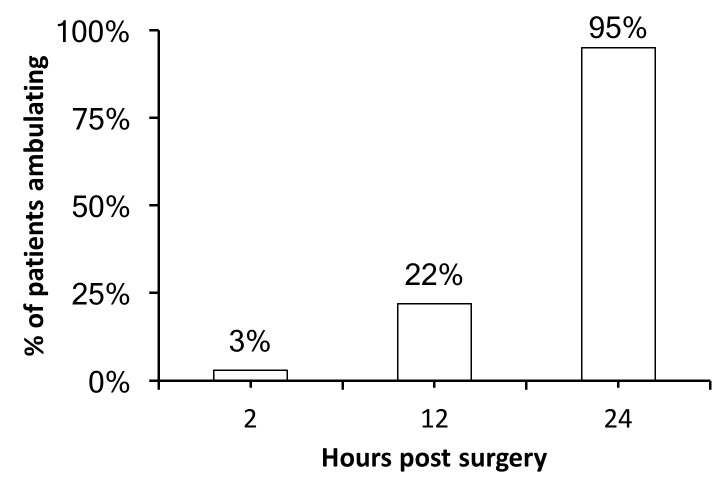
Time to ambulation Percentage of patients who ambulated at different time points after surgery. All patients but two ambulated within 24 hours of surgery.

All patients except two ambulated within 24 hours of the surgery (Figure 4). Of the patients who did not ambulate, one began to ambulate subsequently. The other, who was wheelchair bound before the surgery, still has difficulty ambulating.

### Complications

Of the 36 patients in the study, 31 (86%) were seen at least 150 days post surgery. One patient reported neuropraxia immediately post surgery, which improved at subsequent follow-ups. Nerve irritation with corresponding weakness was found in two patients. In both patients, weakness was 4-5 at the most recent follow-up.

### Pain and Oswestry

Only patients who had pain scale and Oswestry scores recorded before surgery and at least 150 days post surgery were included in the study. The patients’ pain on a ten-point scale decreased from 8.3 ± 1.7 to 3.7±2.7 (N=29 paired Wilcoxon rank sum test p<0.001). The patients' disability on the Oswestry Disability Index was reduced from 53±12% to 32±22% (N=23, paired Wilcoxon rank sum test p<0.001). Seven patients were excluded from Oswestry analysis because they deceased postoperatively or could not be reached for follow-up. Six patients were excluded from Oswestry analysis because they lacked a pre-op Oswestry. Postoperative Oswestry data was available for ﬁve of these patients with a value of 31±22%.

### Radiographic outcomes

Mean Cobb angles decreased from 16.0±6.1° to 9.3±5.8° (paired T-test p<0.001). This result amounts to 2.5° of correction per level of surgery. Detailed imaging was read by independent radiologists for 24 patients at the one year follow-up (minimum 280 days post surgery). In this subgroup of patients, interbody fusion was noted in all cases and all levels. Right- and left-posterolateral fusion was found in 71% and 74% of all levels, respectively. Detailed radiographic outcomes are summarized in Table 3.

**Table 3:** Radiographic outcomes Detailed radiographic outcomes. Cobb angles are mean ± standard deviation. Percentages are of levels operated on unless otherwise indicated.

	Pre-op	Post-op
Cobb Angle	16.0±6.1°	9.3±5.8°
Interbody Fusion	-	100%
Postolateral Fusion Right	-	71%
Postolateral Fusion Left	-	74%
Screw Loosening	-	8.6% (of screws placed)
Screw Breach	-	4.3% (of screws placed)

## Discussion

### Perioperative outcomes

We have previously demonstrated in patients without scoliosis that OLLIF improves perioperative outcomes significantly compared to transforaminal lumbar interbody fusion (TLIF) [15]. Blood loss decreased from 453 to 77 ml, hospital stay from 5.8 to 3.3 days and surgery time from 175 to 107 minutes for a single level TLIF and OLLIF, respectively. Compared to patients without scoliosis, the scoliotic patients in this study had slightly increased blood loss but decreased surgery time (with the exception of three level procedures). The scoliosis cohort may have lower surgery time than the non-scoliosis group because the surgeon had become more familiar with OLLIF over time (learning curve). In general, the perioperative outcomes shown here indicate that the presence of scoliosis does not significantly add to the difficulty of an OLLIF surgery after the surgeon has mastered OLLIF in a non-scoliosis group.

Systematic literature reviews have usually found that minimally invasive surgery is not significantly faster than open surgery and in many cases takes longer [17], which may be due to longer set up time and the multiple images necessitated by some MIS procedures. OLLIF is the first minimally invasive fusion that breaks this trend as it is over 50% faster than an open fusion. One reason for this result may be that OLLIF is designed as a truly minimally invasive procedure, whereas alternatives such as minimally invasive spine transforaminal lumbar interbody fusion (MIS-TLIF) are essentially mini-open procedures where the same approach employed in an open procedure is performed through a smaller incision. As in other MIS fusions, OLLIF substantially decreases hospital stay and blood loss compared to open surgeries.

Except for two patients, all scoliosis OLLIF patients ambulated within 24 hours of surgery. This result represents a major improvement over current open and MIS fusion techniques. Goldstein, et al. (2014) performed a systematic review of the literature on MIS and open lumbar spinal fusion [17]. In the open group, time to ambulation ranged from 2.97-13.4 days compared to 0.9-3.8 days in the MIS group (mean time to ambulation).

The current standard of care for deformity correction involves open surgeries. Mini open approaches clearly document decreased morbidity, shorter hospital stays, and easier recovery without jeopardizing Cobb angle correction [18-21] but have not gained widespread acceptance. Recently, MIS approaches have gained momentum, but their disadvantages include a higher degree of technical difficulty leading to longer surgery time [17, 22-24] and increased complications in the extreme lateral approach [25]. Both of these obstacles have been overcome with the OLLIF technique. We have previously demonstrated that OLLIF is not technically challenging and produces superior results compared to open surgeries [15]. The present study demonstrates that these outcomes are transferable to a group of patients with deformity.

As minimally invasive surgery has become more common, significantly lower infection rates relative to open procedures have been documented. Infection rates range from 0.09%-16% for MIS cases but are usually in the low single digits [26]. In our series of over 500 OLLIF patients to date (including the subset of deformity patients presented here), there have not been any clinically significant infections requiring reopening and drainage. We hypothesize that the lack of infections in OLLIF cases is a major factor in reducing the costs of OLLIF relative to open procedures and we are currently collecting data to test this hypothesis.

### Technical aspects

OLLIF provides stabilization and correction of deformity in scoliosis cases by focusing on segmental treatment of clinically relevant spinal levels, possibly through staged procedures. Only four patients in this group underwent follow-up second stage surgery. Other patients were clinically stable and have not required second stage surgery to date.

The technical advantages of the OLLIF approach include a lack of major blood vessel and organ manipulation when compared to anterior approaches. Compared to the lateral approach, OLLIF is significantly safer in avoiding the lumbar plexus due to the smaller footprint of the instrumentation. Regarding the approachable levels, anterior approaches are mostly designed for L4-S1, modified anterior approaches such as OALIF are best suited for L3-5, lateral approaches are suited only for L1-4 because they are clinically dangerous in L4/5 and obstructed in L5/S1. OLLIF (along with MIS-DLIF), however, can be applied universally from the thoracolumbar junction to S1.

Several studies provide useful evidence to validate one or two components of the OLLIF surgical technique, but there is not a procedure that includes all aspects of OLLIF. Zhu, et al. [3] reported on selective segmental transforaminal interbody fusion combined with posterior-instrumented spinal fusion, with ‘reasonable’ long-term clinical and radiographic outcomes. Further supported papers discuss the advantages of intense curve corrections and easier extension, but worsening adjacent level disease [27-28]. Like these procedures, OLLIF provides 360 degrees of stability but is completely minimally invasive.

There is increasing evidence that an anterior approach and anterior grafting as performed in OLLIF improve the overall outcomes of deformity correction. A meta-analysis of 12 studies on degenerative scoliosis [2] reported greater sagittal and coronal correction in patients receiving anterior support via a lateral lumbar interbody fusion compared to posterior approaches only. The use of open fusion with osteotomy was associated with larger Cobb angle and sagittal balance correction but higher rates of reoperation. Another study compared radiographic outcomes at six weeks for anterior lumbar interbody fusion (ALIF), lateral lumbar interbody fusion (LLIF), transforaminal lumbar interbody fusion (TLIF) and posterior spinal fusion via instrumentation without interbody grafting and found that anterior grafting is critical to correcting lordotic deformity [29]. Finally, Nohara, et al. [1] demonstrated improved correction of scoliosis immediately after surgery for an anterior approach cohort compared to a posterior approach. OLLIF mimics the benefits of anterior approaches as discussed above by eliminating the open posterior component of the procedure.

### Treatment goals

The aim of surgical treatment is to improve the patient’s symptoms. In the case of spinal fusions, this improvement is usually achieved through the decompression of neural elements. Restoration of sagittal and coronal balance can help in achieving symptomatic relief, and we have found that OLLIF can provide substantial correction of deformity. However, the primary aim of OLLIF is not to achieve complete radiographic correction of the spine. Instead, the aim of OLLIF is to improve symptoms by providing decompression and increasing structural support to the anterior and posterior column by in vivo fusion. This factor is an important distinction from traditional treatment goals of scoliosis surgery where radiographic correction is considered the primary outcome measure. However, radiographic correction does not necessarily correlate with clinical symptoms [30]. Here, we focus on the clinical outcomes after an MIS deformity correction and have shown that significant improvements can result.

As an aging population presents with increasing numbers of comorbidities, extensive open surgeries for scoliosis are not an optimal treatment. Many of the patients in this study had been rejected by multiple spine surgeons because of significant co-morbidities. Due to the MIS nature of OLLIF, we were able to treat these patients by choosing spinal levels corresponding to the patient’s clinical symptoms, and balancing the risks and benefits of operating on each level. The data presented in this study prove that OLLIF is a safe and efficacious procedure to treat deformities of the lumbar spine. More importantly, we also show that OLLIF can provide symptomatic relief to patients with deformity and degenerative disc disease who were previously considered ineligible for surgery.

## Conclusions

Oblique lateral lumbar interbody fusion (OLLIF) is a safe and efficacious option to treat degenerative adult deformities even in patients who possess comorbidities. OLLIF employs anterior grafting, which is uniformly supported by the literature to be superior to posterior approaches. However, among other anterior approaches, OLLIF remains extremely under-utilized for adult deformity correction.

Surgeons tend to favor a specific interbody procedure once they are comfortable with that technique. As the learning curve for a new technique is steep, those trained in one specific interbody technique will have a tendency to continue with that technique. For this reason, we strongly recommend including OLLIF in the training of residents in academic institutions and to include this technique among others to consider when performing interbody fusion as it provides significant clinical benefits to the patient.

We are continuing our data collection for this study by increasing the cohort size and continuing long term follow-up with the patients.
